# Effects of livestock grazing on soil, plant functional diversity, and ecological traits vary between regions with different climates in northeastern Iran

**DOI:** 10.1002/ece3.5396

**Published:** 2019-06-27

**Authors:** Soroor Rahmanian, Martin Hejda, Hamid Ejtehadi, Mohammad Farzam, Farshid Memariani, Petr Pyšek

**Affiliations:** ^1^ Department of Biology, Faculty of Sciences Ferdowsi University of Mashhad Mashhad Iran; ^2^ Department of Invasion Ecology, Institute of Botany The Czech Academy of Sciences Průhonice Czech Republic; ^3^ Department of Range and Watershed Management, Faculty of Natural Resources and Environment Ferdowsi University of Mashhad Mashhad Iran; ^4^ Department of Botany, Research Center for Plant Sciences Ferdowsi University of Mashhad Mashhad Iran

**Keywords:** functional diversity, grazing, Iran, precipitation, soil fertility, species traits

## Abstract

Understanding the responses of vegetation characteristics and soil properties to grazing in different precipitation regimes is useful for the management of rangelands, especially in the arid regions. In northeastern Iran, we studied the responses of vegetation to livestock grazing in three regions with different climates: arid, semiarid, and subhumid. In each region, we selected 6–7 pairwise sampling areas of high versus low grazing intensity and six traits of the present species were recorded on 1 m^2^ plots—five grazed and five ungrazed in each area. The overall fertility was compared using the dissimilarity analysis, and linear mixed‐effect models were used to compare the individual fertility parameters, functional diversity indices, and species traits between the plots with high and low grazing intensity and between the climatic regions. Both climate and grazing, as well as their interaction, affected fertility parameters, functional diversity indices, and the representation of species traits. Grazing reduced functional evenness, height of the community, the representation of annuals, but increased the community leaf area. In the subhumid region, grazing also reduced functional richness. Further, grazing decreased the share of annual species in the semiarid region and seed mass in the arid region. Larger leaf area and seed mass, smaller height and lower share of annuals were associated with intensive grazing. Species with large LA and seed mass, lower height and perennials can be therefore presumed to tolerate trampling and benefit from high nutrient levels, associated with intensive grazing. By providing a detailed view on the impacts of overgrazing, this study highlights the importance of protection from grazing as an effective management tool for maintaining the pastoral ecosystems. In general, the composition of plant traits across the pastures of northeastern Iran was more affected by intensive grazing than by the differences in climate.

## 1 INTRODUCTION

Livestock grazing represents the dominant land use in grasslands across the world, where it forms the economical basis of local welfare (Kelaidis, 2015). Grazing by livestock affects plant growth, community structure, ecosystem functioning, and services in grasslands worldwide (Díaz et al., 2007; Lemaire, Hodgson, Moraes, Carvalho, & Nabinger, 2000). Therefore, several reviews have summarized the effects of grazing on vegetation and soil conditions and suggest that climate interacts with grazing history in their effects on grasslands (McSherry & Ritchie, 2013; Milchunas & Lauenroth, 1993; Milchunas, Sala, & Lauenroth, 1988; Török, Hölzel, Diggelen, & Tischew, 2016).

Intensive grazing is therefore also likely to modify the representation of species ecological traits within the grazed communities (Tóth et al., 2018; Zheng, Li, Lan, Ren, & Wang, 2015). A better understanding of mechanisms underpinning the plant responses to grazing and their linkages to ecosystem functioning (mediated by the resource availability) is fundamental for the conservation and sustainable management of pastoral ecosystems (Zheng et al., 2015). Grazing usually increases the dominance of species with resistance strategies in dry and infertile environments, and the dominance of species with tolerance strategies in the humid and fertile environments (Coley, Bryant, & Chapin, 1985; Herms & Mattson, 1992). This traditional assumption states that resistance and tolerance are two alternative strategies of adaptation to intensive grazing (Van der meijden, Wijn, & Verkaar, 1988). Therefore, in the environments with little, erratic rainfall, the effects of climate and grazing on vegetation and soil can be rather complex (Cheng et al., 2011).

For soils, plants are the main contributors of organic carbon (SOC) and they also determine the quality and quantity of litter and roots (McSherry & Ritchie, 2013). Livestock grazing affects the soil conditions by direct mechanical disturbance, such as trampling, and nutrient addition from dung and urine inputs (Schrama et al., 2013).

Functional characteristics of plants directly or indirectly affect their survival, growth, and reproduction (Violle et al., 2007). For this reason, approaches based on functional traits have come out as a promising way to understand plant ecological strategies, plant‐herbivore interactions, and their linkages to ecosystem functioning (De Bello, LepŠ, & Sebastià, 2005; Violle et al., 2007; Zheng et al., 2015). Functional traits make it easier to understand the mechanisms how the plant community responds to environmental gradients (Funk et al., 2017; Lavorel & Garnier, 2002; Zheng et al., 2015). The method of “community‐weighted means” is one of the most common methods for analyzing the trait–environment relationships (Ricotta & Moretti, 2011). The values of traits of species present in the community are used to calculate a mean value, characteristic for each trait and sample, by averaging the values of individual species, weighted by their relative abundances (Lepš, Bello, Šmilauer, & Doležal, 2011). On the contrary, the overall distribution of trait values in a community can be expressed by several measures of FD, which reflects the variability in traits (Díaz et al., 2007; Laliberté & Legendre, 2010; Mason & de Bello, 2013).

The arid, semiarid, and subhumid grasslands of northeastern of Iran and the Middle East in general have experienced a long history (>4,000 years) of livestock grazing (Beck, 1998; Farzam & Ejtehadi, 2016; Jankju, 2016). Although there is a large body of studies on plant responses to grazing worldwide, focusing mostly on species richness and diversity (Ganjurjav et al., 2015; Herrero‐Jáuregui & Oesterheld, 2018; Proulx & Mazumder, 1998; Zhu, Jiang, & Zhang, 2016), only a few studies were conducted in Iran (e.g., Jafarian, Kargar, Tamartash, & Alavi, 2019; Moradi & Oldeland, 2019), especially in the northeast of this country (e.g., Jafari, Zarre, Alavipanah, & Ghahremaninejad, 2015). Furthermore, most of the current knowledge of plant‐trait responses to grazing (see Briske, 1996 for a review) is based on local studies under specific environmental settings. In our study, we compare the effects of grazing between contrasting climates, which represents a major novelty in the context of the current literature on grazing (Díaz et al., 2007; Pakeman et al., 2008; Wang et al., 2017). Moreover, we use an approach based on functional traits, a methodology that has become increasingly popular recently, as it provides a better understanding of the effects of grazing and associated mechanisms (Danet, Kéfi, Meneses, & Anthelme, 2017; Díaz, Noy‐Meir, & Cabido, 2001; Vesk & Westoby, 2001).

Our research therefore aimed to investigate the relative effect of grazing and its interaction with rainfall on soil fertility, plant functional diversity, and the representation of traits within a community expressed as the community‐weighted means. We addressed the following questions: (a) Does the climate, grazing, and the interaction between these factors affect soil fertility? (b) Does the climate, grazing, and the interaction between these factors affect the functional diversity of plant communities? and (c) Does the climate, grazing, and the interaction between these factors affect the representation of different ecological characteristics, represented by the community‐weighted means?

## 2 METHODS

### 2.1 Study area and sampling

**Figure 1:**
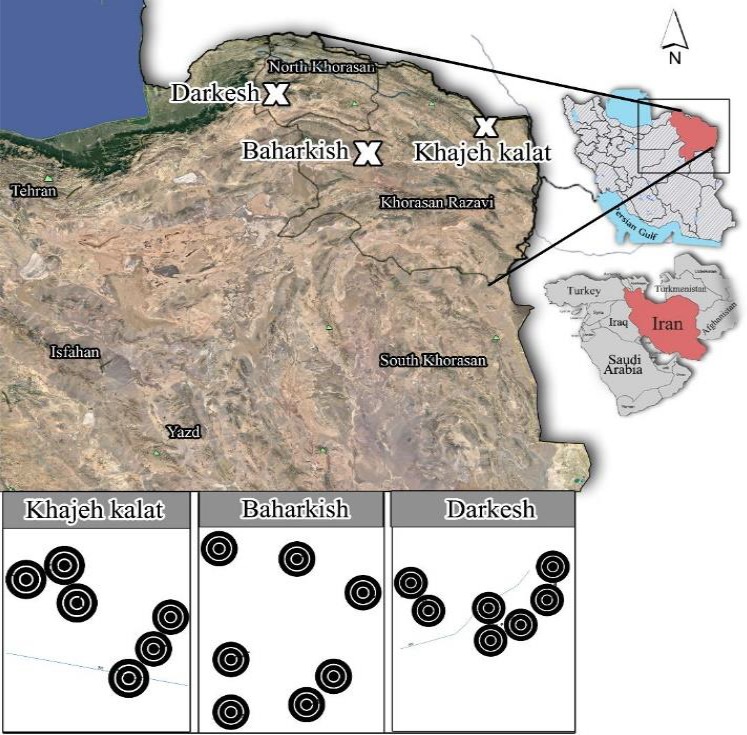
Map of the study area in NE Iran, showing the Khajeh Kalat as arid region with approximately 255 mm of annual precipitation; Baharkish rangeland as semiarid region with approximately 354 mm of annual precipitation and Darkesh as subhumid region with approximately 550 mm annual of precipitation (see Figure 3 for more details on the sampling area). The circles represent individual sampling areas in each region

**Figure 2:**
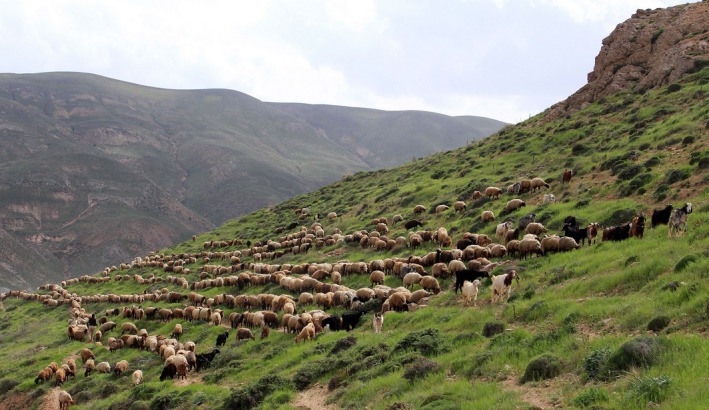
High grazing intensity in the semiarid region, grazed by sheep and goats. Photograph by Vahid Jafari

We selected three regions with different precipitation in northeastern Iran, in the Khorassan‐Kopet Dagh floristic province of the Irano‐Turanian region (Figure 1; Table 1). Climatic parameters ranged from arid to subhumid. There are sharp gradients of livestock grazing, ranging from a very high to very low intensity, all within small and therefore relatively homogenous areas. Further, there are very heterogenous climatic conditions, with a mean annual rainfall varying from less than 250 to more than 550 mm per year. The target area is inhabited by ethnically and culturally homogenous human population (nomads of the Kormanj Tribes), who apply similar methods of livestock utilization: they keep sheep and goats from early March to late July (Figure 2). Although different grazing animals differ in their effects on vegetation (Tóth et al., 2018), sheep and goat have similar impact; the animals are of similar size and comparable in their ability to pick plant parts such as flowers, pods, and young shoots (Celaya et al., 2003; Oliván & Osoro, 1998). Further, there were similar proportions of sheep and goat across all three climatic regions. In all three climatic regions, we selected homogenous areas with two levels of grazing intensity (low‐intensity grazing = 0.5 animal units per month and hectare, further termed AUM; high intensity = 2–3 AUM/ha; see Table 2 for details).

**Table 1:** Basic characteristics of the study area

Location	Zone	Coordinates	Elevation (m)	Mean annual precipitation
Khawjeh Kalat	Arid	60°27′–60°34′E, 35°43′–35°50′N	630–810	255
Baharkish	Semiarid	58°40′–58°36′E, 36°44′–36°42′N	1,580–2,390	354.4
Darkesh	Subhumid	56°43′–58°56′E, 37°23′–37°26′N	1,160–1,660	550

Note

Data were obtained from Iranian Meteorological Organization (data from 1996–2009).

**Table 2:** General description of grazing history in the arid, semiarid, and subhumid climatic region in northeastern Iran

Climate	Type of grazing	Type of grazers	Grazing intensity	Grazing history and management	Density of grazers
Arid	Seasonal, 20 March–10 May	Sheep (90%), Goat (10%)	High grazed	Seasonal‐free ranging	3 AUM/ha
			Low grazed	Protected in the last 35 years, occasional light grazing in some years	0–0.5 AUM/ha
Semiarid	Seasonal, 20 May–23 July	Sheep (90%), Goat (10%)	High grazed	Seasonal‐free ranging	2 AUM/ha
			Low grazed	Protected in the last 35 years, occasional light grazing in some years	0–0.5 AUM/ha
Subhumid	seasonal, 5 May–15 July	Sheep (85%), Goat (15%)	High grazed	Seasonal‐free ranging	2.5 AUM/ha
			Low grazed	Protected in the last 37 years, occasional light grazing in some years	0–0.5 AUM/ha in some years

The arid region, located in the eastern Kopet Dagh, is a steppe rangeland. The area has a dry climate, with mean annual precipitation of 255 mm. The semiarid region is also a steppe rangeland in the eastern part of Khorassan‐Kopet Dagh. The area has mild and dry summers but cold and wet winters, with the mean annual precipitation (20‐year data) of 354 mm. The subhumid region is a forest steppe, located in the western part of Khorassan‐Kopet Dagh. The mean annual precipitation is 550 mm, with the highest precipitation from late autumn to early spring and with a summer drought.

**Figure 3:**
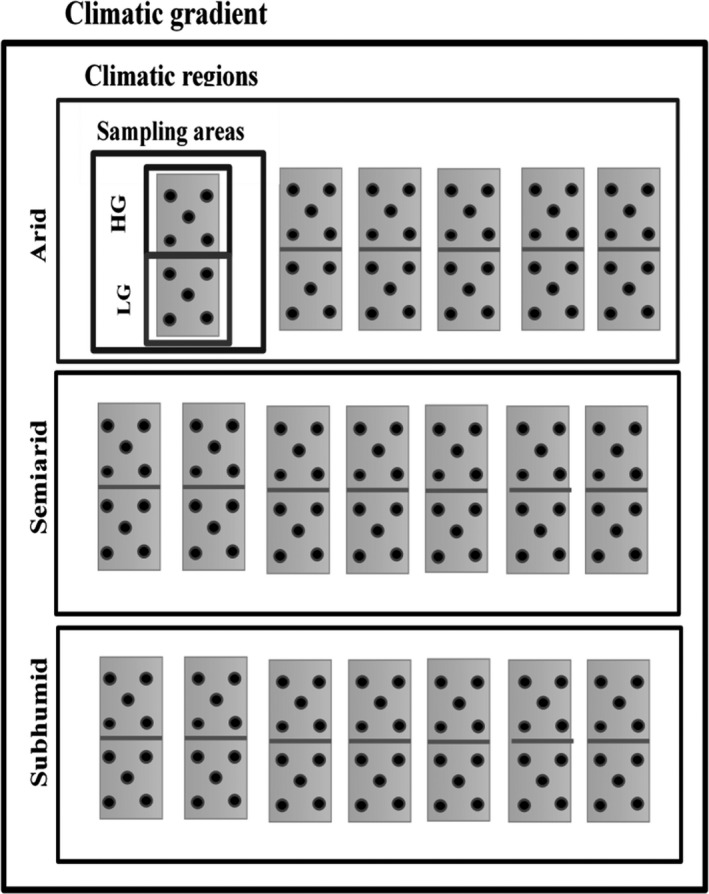
Scheme of the sampling design. We recorded all species present in the individual plots of 1 × 1 m, with five HG and five LG plot present in each sampling area, separated by fence. The sampling areas were considered as homogenous units and independent replicates within each different climatic regions, based on the annual sum of precipitation (arid, semiarid, and subhumid). HG, high grazing; LG, low grazing

The sampling design was arranged in a hierarchical way, with 6–7 individual sampling areas nested in each of the three climatic regions (arid, semiarid, and subhumid) and with five high grazing intensity plots (HG) and five low grazing intensity plots (LG), nested in each of the sampling areas (see Figure 3 for more details on the sampling design). Altogether, 200 plots were sampled: three climatic regions, six sampling areas in the arid, seven sampling areas in the semiarid, and seven in the subhumid region, 10 plots (five HG and five LG) in each sampling area. The mean size of sampling areas was 1,600 ha in the arid, 1,035 ha in the semiarid and 2,000 ha in the subhumid region. The mean distance between the individual sampling areas was 1.63 (±0.51) km and the minimal distance between two independent sampling areas was 1 km. Within each of the sampling areas, the individual HG/LG plots (with an area of one square meter) were placed randomly, in a relatively homogeneous area in terms of topography, land use and vegetation. The LG plots were located within fences that have prevented grazing for around 35 years, whereas HG plots were open and therefore have suffered from a long‐term overgrazing. Each plot was characterized by its geographic coordinates and altitude. In 2017, the cover (%) of all present plant species was recorded between April and June, when the growing season peaks in this region.

The decision about the grazing status of the plots (HG vs. LG) was supported by the observed median number of dung deposits: 55.3 in the HG and 11.7 in the LG plots and also by the width of the microterrace livestock paths in the horizontal way: (0.27 ± 0.09) m for the HG plots and (0.04 ± 0.04) for the LG plots. The low amount of grazing on the LG plots is due to goats, which can climb over the fences and it was therefore difficult to eliminate them from the LG plots completely.

### 2.2 Soil collection and processing

Two soil samples (0–15 cm depth) were collected at each sampling area from the HG and LG plots, after the aboveground material had been harvested. Soil was collected from each plot using a bucket auger and then mixed into a single soil sample (for the HG and LG plots separately). All of the soil samples were brought into the laboratory in airtight plastic bags. All of the soil samples were air‐dried and then filtered through a 0.2 mm sieve, discarding the visible roots and other plant debris. Soil pH and electrical conductivity (EC) were measured using a pH meter and a conductivity meter in saturated mud. Soil organic carbon and soil organic matter (OM) were determined using the Walkley–Black method (Nelson & Sommers, 1982). The soil available nitrogen (N), phosphorus (P), and potassium (K) were measured using the methods of Miller and Keeney (1982).

### 2.3 Data management

#### 2.3.1 Species diversity indices

We recorded six traits of 337 species, following Peìrez‐Harguindeguy et al. (2013): height, leaf area, seed mass, clonality, and life history. These traits have been described as indicators of plant dispersal, establishment, persistence, and response to grazing (Díaz et al., 2007; Weiher et al., 1999) and represent an extension of the LHS scheme (leaf, height, seed traits; Westoby, 1998; see also Hejda & de Bello, 2013). We used leaf area rather than specific leaf area, because the specific leaf area was not available for many of the recorded species. The seed mass data come from the Royal Botanic Gardens Kew's Seed Information Database (www.rbgkew.org.uk/data/sid/). Plant height is the shortest distance between the upper foliage boundary and ground level. Leaves were scanned to measure leaf area with digital photo in the field with a scale bar. Then, we used the J image software (Glozer, 2008) to calculate leaf areas. Clonality was expressed as the presence/absence of clonal reproduction of individual species, be it via rhizomes or runners. Effects of functional traits on ecosystem properties have been quantified by the method of community‐weighted means (CWM—Garnier et al., 2004; Lavorel & Garnier, 2002) and functional diversity (FD—Mason & de Bello, 2013). CWM traits are calculated as mean trait values for each vegetation plot, weighted by the relative abundances of species with that particular trait values (Shipley, Vile, & Garnier, 2006; Zhu et al., 2016). The functional diversity of each plot was expressed using the three different, yet complementary indices: functional richness (FRich), determined by the occurrences of species and therefore independent on species' abundances but related to species richness; functional evenness (FEve), expressing the evenness of the trait values; and functional divergence (FDiv), expressing the divergence in the distribution of functional traits (see Villéger, Mason, & Mouillot, 2008 or Mouchet, Villéger, Mason, & Mouillot, 2010 for more details). We computed a matrix of species functional dissimilarities using Gower distance and used it to calculate FD (Rao, 1982). In this way, FD is the sum of dissimilarities (or distances) in trait space between all possible pairs of species, weighted by the species' relative abundances.

The community‐weighted means (CWM) for each trait and community sample were calculated as Σ*P_i_* × Trait *i*, where *P_i_* is the relative abundance of species “*i*” in the community sample and *j* trait *i* is the trait value. Further, mean values of individual traits (height, seed mass, leaf area, clonality, annual–perennial life history) were calculated for each vegetation plot. Eventually, the mean trait values per plot (weighted by the relative abundances of species) were used as importance values in the analyses.

### 2.4 Statistical analyses

**Figure 4:**
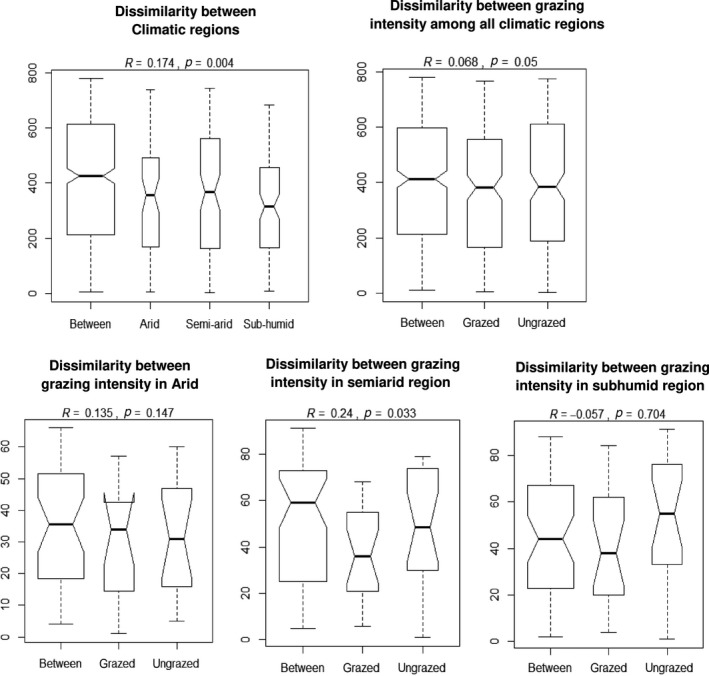
Using the permutation tests, the analysis of similarities provides a way how to test the differences between the climatic regions, grazing regimes as well as the interaction of these factors. If the groups of sampling units differ in their soil fertility, then compositional dissimilarities between the groups are larger than those within the groups (999 permutations). The figure shows there are significant differences in Bray–Curtis dissimilarities, expressing the internal heterogeneity, between the three climatic regions. On the contrary, the heterogeneity between the HG and LG plots is significant for the semiarid climatic region only. HG, high grazing; LG, low grazing

We used the function “anosim” (analysis of similarities) of the “vegan” package of the R software, to measure the Bray–Curtis dissimilarities in soil fertility between the HG and LG plots as well as between the different climatic regions (Figure 4). We used permutation tests (999 permutations—Permutational Analysis of Multivariate Dispersions) to test the significance of the differences between the climatic regions, between the HG versus LG plots as well as the interaction of these two factors. Then, the differences in soil fertility parameters, functional diversity indices, and species traits (expressed as the CWM values) between the HG and LG plots and between the three climatic regions were tested using the linear mixed‐effect models, with “sampling areas” as random effect (nested in “climatic region”), “climatic region” and “grazing” as fixed effects and functional diversity (functional richness, functional evenness, and functional divergence), community‐weighted means of trait values (height, seed mass, leaf area, clonality, annual—perennial life history), and soil fertility parameters (pH, EC, N, P, K, OC, and OM) as response variables. All univariate analyses were performed in the R software (R Development Core Team, 2014), using the NLME package. The script for the model testing the main effect of climate and the interaction between “climatic region” and “grazing” was “lme (response variable − climatic region/grazing, random = −1|sampling area).” The main effect of grazing was tested in separate models, with “climatic region” and “sampling areas” (nested in “climatic region”) as random effects. The script of the models testing the main effect of grazing was “lme (response variable − grazing, random = −1|climatic region/sampling area). The normality of the input data was assessed based on Shapiro–Wilk tests, and the normality of residuals was checked visually, by plotting the observed values against the fitted values. CWM values were log‐transformed (leaf area, plant height, seed mass) for the purpose of the box‐plots to reduce the impacts of outliers. CWM for each trait and FD indices for all traits in combination were calculated using the FD package (Laliberté & Legendre, 2010) in the “R” software, version 3.1.1 (R Development Core Team, 2014).

## 3 RESULTS

### 3.1 Effect of climate, grazing, and their interactions on soil fertility

As shown by the permutation tests with Bray–Curtis dissimilarities, the soil fertility differs significantly between the climatic regions (*p* = 0.004), as well as between the HG–LG plots (*p* = 0.05). However, the differences between HG and LG plots were only significant in the semiarid region (Figure 4).

The LME model results demonstrate that most of the measured soil fertility parameters (available soil K, OM, and OC) differed significantly between the climatic regions. On the other hand, no significant main effect of grazing on soil fertility parameters was detected, with the exception of K; larger values of this element were found in HG plots. The climate × grazing interaction was nonsignificant for soil fertility, except for the K content (Table 2).

### 3.2 Effect of climate, grazing, and their interactions on functional diversity

No significant differences in functional diversity were detected between the three climatic regions (Table 4). Out of the functional diversity indices (functional richness, functional evenness, and functional divergence), only functional evenness differed significantly between the HG and LG plots (Table 4), with the LG plots showing larger values, compared to the HG plots (Table 3).

**Table 3:** The mean values and standard errors of species traits (represented by the CWM values), FD indices (functional richness, evenness, and divergence) and soil fertility parameters (soil Potassium, organic matter content, organic Carbon) according to the factors of climate and grazing

	Responses	Main effect of grazing in all three climatic regions		Main effect of climate on both HG and LG plots		
		HG	LG	Arid	Semiarid	Subhumid
Community‐weighted means trait	CWM‐plant height	53.8 ± 3.09	72.57 ± 5.44	60.56 ± 4.18	57.05 ± 1.28	71.57 ± 7.34
	CWM‐leaf area	1,021.13 ± 198	564.47 ± 62.6	557.37 ± 123	705.53 ± 149.2	1,081.85 ± 234.86
	CWM‐seed mass	350.85 ± 227.21	141.17 ± 50.2	278.1 ± 88.38	10.97 ± 7.08	453.55 ± 322.85
	CWM‐clonality	6.73 ± 2.46	11.07 ± 4.7	10.05 ± 3.89	2.03 ± 1.49	14.79 ± 6.57
	CWM‐annual	46.65 ± 5.45	79.89 ± 8.12	55.29 ± 7.57	56.64 ± 6.07	76.75 ± 11.16
	CWM‐perennial	5.88 ± 2.34	8.66 ± 3.2	8.016 ± 4.2	11.79 ± 4	2.11 ± 1.63
Functional diversity	Functional richness	70.88 ± 3.41	76.28 ± 2.92	75.88 ± 4.24	76.45 ± 3.82	68.74 ± 3.65
	Functional evenness	0.59 ± 0.015	0.63 ± 0.01	0.63 ± 0.018	0.63 ± 0.01	0.58 ± 0.01
	Functional divergence	0.79 ± 0.012	0.8 ± 0.009	0.77 ± 0.016	0.8 ± 0.01	0.79 ± 0.01
Soil fertility	EC	0.64 ± 0.07	0.82 ± 0.16	0.86 ± 0.22	0.6 ± 0.07	0.76 ± 0.17
	pH	8.08 ± 0.08	8.13 ± 0.12	8.21 ± 0.09	8.09 ± 0.12	8.04 ± 0.18
	Phosphorus(mg/100 g)	11.95 ± 0.57	11.44 ± 0.73	12.48 ± 1.06	11.63 ± 0.63	11.08 ± 0.81
	Nitrogen (mg/100 g)	0.2 ± 0.06	0.22 ± 0.08	0.15 ± 0.06	0.21 ± 0.07	0.26 ± 0.12
	Potassium (mg/100 g)	256.25 ± 1.06	242.5 ± 1.16	231.5 ± 1.36	267.28 ± 1.44	246.78 ± 1.16
	Organic Matter (g/100 g)	2.82 ± 0.24	3.04 ± 0.33	1.6 ± 0.21	3.01 ± 0.3	3.99 ± 0.44
	Organic Carbon (g/100 g)	1.64 ± 0.19	1.76 ± 0.25	0.92 ± 0.16	1.75 ± 0.21	2.33 ± 0.3

Abbreviations: HG, high grazing; LG, low grazing.

**Figure 5:**
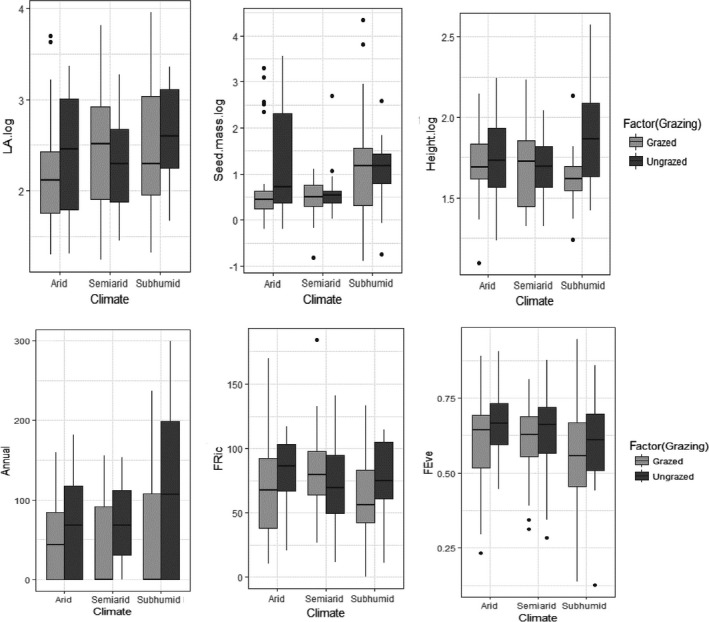
The community weighted means of individual traits (logarithm of LA, logarithm of seed mass, logarithm of height, annual life form) and functional diversity between the HG and LG plots and across the three climatic regions. HG, high grazing; LG, low grazing

The LME models also show that the differences between the HG and LG plots significantly vary with climate only for the functional richness, with larger values on the LG plots in the arid and subhumid regions, but not in the semiarid region (Figure 5; Table 4).

**Table 4:** Results of linear mixed‐effect models on the effects of climate, grazing and the climate × grazing interaction on the representation of species traits within the community (CWM values), FD indices and soil fertility parameters (soil Potassium, organic matter content, organic Carbon)

Predictors		Responses	*df*	*F*	*p*‐Value
Climate	Soil fertility	Available soil potassium (mg/100 g)	17	8.09	**0.003** **
		Organic matter content (g/100 g)	17	8.73	**0.002** **
		Organic carbon	17	8.87	**0.002** **
Grazing	Community‐weighted means trait	CWM‐plant height	179	10.66	**<0.001** ***
		CWM‐leaf area	179	5.327	**0.022** *
		CWM‐annual	179	11.74	**8e−04** ***
	Functional diversity	Functional evenness	179	5.25	**0.0231** *
	Soil fertility	Available soil potassium (mg/100 g)	19	5.57	**0.0291** *
Climate × grazing	Community‐weighted means trait	CWM‐plant height	177	12.09	**0.0001** ***
		CWM‐leaf area	177	2.85	**0.038** *
		CWM‐seed mass	177	3.54	**0.015** *
		CWM‐annual	177	4.64	**0.003** **
	Functional diversity	Functional richness	177	5.04	**0.002** **

Note

Significant terms are in bold. All combinations were tested and only significant results are presented.

‐lme (response variable − climate/grazing, random = −1|sampling area).

‐lme (response variable − grazing, random = −1|climate/sampling area).

* *p* < 0.05,

** *p* < 0.01,

*** *p* < 0.001

### 3.3 Effect of climate and grazing and their interactions on species traits

No significant differences in the recorded species traits (expressed as the CWM values) were detected between the three climatic regions (Table 4). In contrast, the CWM values for height, LA, and the share of annual species significantly differ between the HG and LG plots (Table 4), with the HG plots having fewer annuals and larger values of the leaf area. On the contrary, the mean plant height was larger on the LG plots, compared to the HG plots (Table 3).

The significant interaction between climate and grazing shows that the differences between the HG and LG plots vary between the three climatic regions for plant height, LA, seed mass, and the representation of annuals (Figure 5; Table 4). The LG plots revealed higher values for height in the subhumid region; however, the differences between the HG and LG plots were not apparent in the other two climatic regions. Also, no differences were detected between the HG and LG plots for the seed mass in the semiarid and subhumid regions, however, larger values for the LG plots were detected in the arid region. The leaf area showed larger values for the LG plots in the arid region, however, only small differences between the HG and LG plots were detected in the semiarid and subhumid regions. The representation of annual species was higher on the LG plots in the semiarid and subhumid regions, while there was no apparent difference between the HG and LG plots in the arid region (Table 3; Figure 5).

## 4 DISCUSSION

### 4.1 Effects of climate, grazing, and their interaction on soil fertility

Climate was detected to have a significant effect on soil fertility (Table 3). The results demonstrate that most of the measured soil fertility parameters (available soil K, OM, and OC) differed significantly between the climatic regions. The available soil OM and OC tended to decrease with aridity, as the subhumid region can accumulate a large amount of soil organic carbon and soil organic matter, because of more foliage (Schuur et al., 2001). On the contrary, the highest values of K were recorded in the semiarid region (Table 3). The overall soil fertility differed significantly between HG and LG plots, but the effect was negligible except for K, the larger values of which were measured in HG plots. The higher levels of soil K in the HG plots may be related to trampling by livestock and to the accumulation of animal excrements (Garcia, Sampaio, & Nahas, 2011; Javadi, Jafari, Azarnivand, & Zahedi, 2006; Kohandel, Arzani, & Hosseini, 2006; Zarekia, Jafari, Arzani, Javadi, & Jafari, 2012). The levels of soil K may also increase due to the lower vegetation cover in the HG plots. On the contrary, the climate × grazing interaction was not significant for soil fertility. Therefore, the soil is likely to be affected more by the climate rather than by the grazing intensity.

### 4.2 Effects of climate, grazing, and their interaction on functional diversity and representation of species' characteristics

We did not detect any significant differences in FD indices and the CWM values between the three climatic regions. Even though the extreme arid conditions have been shown to affect the FD (de Bello, Lepš, & Sebastià, 2006) and species traits (De Bello et al., 2006; Wang et al., 2017), such effect was not observed in our study system. This may be due to the fact that our study system did not really include extremely dry conditions, even in the “arid” region.

On the contrary, grazing had a significant effect on functional evenness and trait values, expressed by the CWM method. The results show that plant height and the representation of annuals revealed lower values on the HG plots, with the most pronounced differences between the HG and LG plots in the subhumid region. The subhumid region receives a stable and sufficient precipitation, so plant‐herbivore interactions might be the main driver of vegetation dynamics (see also Lauenroth & Burke, 2008; Milchunas, Lauenroth, Burke, & Detling, 2008). In other words, the suppressive effect of grazing on annuals was the strongest in the subhumid conditions, where the diversity is unlikely to be limited by the drought. This may be also due to the high share of endemics in this region, including annuals, which mostly rank among the well‐palatable species. Livestock has a selective pressure and annuals tend to be relatively more palatable and readily eaten by the grazing livestock (Mosley & Roselle, 2006). Therefore, the grazing‐induced replacement of species (perennials instead of annuals) occurs as a result of the selective utilization of plant species on the HG plots in the subhumid region. In this situation, most species are adapted to competition, rather than to the stress and disturbances induced by the intensive grazing and trampling (see also Craine, Fargione, & Sugita, 2005; Lezama & Paruelo, 2016; Stevens, Reese, & Connelly, 2012). On the contrary, steppes in the arid and semiarid regions of northeastern Iran differ in their flora and vegetation, with most species adapted to drought and herbivory—by possessing toxic compounds or spiny leaves and stems. Therefore, the differences between the share of annuals on the HG versus LG plots are less pronounced in the arid and semiarid region. The values of the leaf area were larger on the HG plots. This likely represents the tolerance‐of‐grazing strategy, as species with large LA and therefore rapid leaf turnover regrow their leaves quickly after grazing (An & Li, 2014; Westoby, Eldridge, & Freudenberger, 1999).

The functional richness was significantly affected by the interaction between grazing and climate as well: the LG plots showed higher functional richness than the HG plots in the arid and subhumid region but not in the semiarid region, which is consistent with other studies (Díaz et al., 2001; Zheng et al., 2011). In the subhumid region, the LG plots revealed higher values of height, however, the difference between the HG and LG plots was not apparent in the other two regions. Annuals were suppressed by the intensive grazing in the semiarid and subhumid regions, where the relatively rich precipitation allows for a high diversity of annuals, which also benefit from more time for growth, compared to the dry climatic region (Peppe et al., 2011). Interestingly, no differences in the representation of annuals between the HG and LG plots were detected in the arid region. Concerning the representation of annuals, the establishment of short‐lived species strongly depends on precipitation. The intensive grazing apparently becomes the limiting factor in relatively favorable conditions, where annual species are not limited by the precipitation. Annuals growing in favorable conditions also tend to have larger leaves and are generally more palatable to livestock. The values for the seed mass were larger on the LG plots in the arid region, however, no differences were detected between HG and LG plots in the other two regions. The larger seeds provide an advantage for germination as well as growth and survival of the seedlings in suboptimal environments (Golodets et al, 2009), possibly acting as a buffer against poor environmental conditions (Pakeman et al., 2008), especially when protected from grazing. On the contrary, small seeds enhance the chances of dispersal to suitable conditions (Baskin & Baskin, 1998), such as small gaps of open soil. Trampling by domestic animals creates such patches, thus increasing germination opportunities, especially for small‐seeded plants (Kahmen & Poschlod, 2008). The leaf area was larger on the LG plots in the arid region, however, no differences between the HG and LG plots were detected in the semiarid and subhumid regions. Although the grand mean for CWM‐LA was larger in the HG plots, the pattern was reversed in the arid region where LG plots had greater CWM‐LA value. This may be due to the grazers usually preferring larger leaves in arid regions, where most plants have small leaves. Then, the mean LA within the community can be expected to decrease as a result of an intensive grazing pressure in the arid region (Díaz et al., 2001; Landsberg, Lavorel, & Stol, 1999).

Grazing suppressed the diversity of species, consistently across the three climatic regions (Rahmanian et al., unpublished data, Table 5). This suggests that grazing is a significant factor, limiting species diversity across the whole study system (see also Osem, Perevolotsky, & Kigel, 2002; Erfanzadeh, Omidipour, & Faramarzi, 2015; Herrero‐Jáuregui & Oesterheld, 2018). Most importantly, we found a highly significant interaction between the effect of climate and grazing on species diversity, with the most pronounced differences between the HG and LG plots detected in the subhumid climatic region (Rahmanian et al., unpublished data, Table 5), where the diversity is unlikely to be limited by the drought. However, the values of species richness and Shannon index revealed different patterns, compared to functional diversity. The FD, for example, did not differ between the HG/LG plots except for the functional evenness. Out of the FD indices tested, a significant interaction between climate and grazing was detected for functional richness only, with the most pronounced difference between the HG and LG plots detected in the subhumid region (Figure 4). This shows that in general, the functional diversity and species diversity can act independently on each other. Further, the suppressive effect of intensive grazing on species diversity in all three climatic regions was not followed by similar trajectories for functional traits. Of course, high intensity of grazing changes the representation of species traits within the community. For example, species with larger leaf area and seed mass were more represented on the grazed plots. This also reflects that plants adopt different strategies against livestock grazing to avoid it or to tolerate it (Díaz et al., 2001). Therefore, the lower species diversity on the HG plots was not mimicked by similar patterns of functional diversity (with all traits together; see for example Li et al., 2015).

**Table 5:** (a) Mean (±*SD*) values of species richness and species diversity (expressed as the Shannon H), recorded on the HG versus LG plots and in each of the three climatic regions. (b) Results of linear mixed‐effect models, testing the effects of climate, grazing and the climate × grazing interaction on species richness (numbers of species) and species diversity (Shannon H)

	(a)	Shannon H (mean value)	Species richness (mean value)
Climate (both HG and LG plots)	Arid	1.53 ± 0.34	10.3 ± 2.7
	Semiarid	1.8 ± 0.32	10.3 ± 2.84
	Subhumid	1.65 ± 0.53	10.58 ± 4.56
Grazing (in all climatic regions)	HG	1.44 ± 0.41	8.71 ± 2.93
	LG	1.87 ± 0.4	12.06 ± 3.6
HG and LG plots (within each of the climatic regions)	Arid‐HG	1.35 ± 0.33	8.53 ± 2.8
	Arid‐LG	1.72 ± 0.36	12.06 ± 2.55
	Semiarid‐HG	1.62 ± 0.35	9.14 ± 2.6
	Semiarid‐LG	1.97 ± 0.3	11.45 ± 3.1
	Subhumid‐HG	1.36 ± 0.55	8.48 ± 3.4
	Subhumid‐LG	1.94 ± 0.52	12.68 ± 5.14

	(b)	*F*	*p*‐Value	*F*	*p*‐Value
LME model	Climate	1.27	0.3	0.03	0.95
	Grazing	86.97	<0.001***	70.48	<0.001***
	Climate × Grazing	31.2	<0.001***	25.12	<0.001***

Abbreviations: HG, high grazing; LG, low grazing.

*** *p* < 0.001

Also, the trait‐based response of the community, represented by the CWM values, can differ substantially from the FD indices, as the responses of individual traits may be more sensitive than multi‐trait responses, expressed by the FD indices (Li et al., 2017). As a result, intensive grazing reduced species diversity, but only some of the FD indices. This suggests that applying different indices of diversity provides the advantage of a more detailed view on the effects of grazing and its interaction with different climatic regimes.

## 5 CONCLUSION

Our data highlight that climate, ranging from arid to subhumid (as represented by the precipitation), appears to be more important than grazing pressure for soil fertility. The responses of soil tend to be slower than the responses of vegetation and thus represent the long‐term effect (Tanentzap & Coomes, 2012). In contrast, grazing was a more important predictor for FD indices and species traits across northeastern Iran, probably because of the combined effects of mechanical disturbance and nutrient input from livestock. Grazing decreased the functional richness and the mean plant height (by reducing the growth of plants and by excluding tall species from the HG communities) in the subhumid region only, where tall plants and especially annuals are apparently more sensitive to the grazing pressure. The grazers are usually regarded as disturbance generators, because they consume leaves and fruits and induce substantial disturbance by trampling (Crawley, 1996). This research increases our knowledge on the responses of species with different functional traits to grazing in different climatic regimes, ranging from arid to subhumid.

## CONFLICT OF INTEREST

None declared.

## AUTHOR CONTRIBUTIONS

H.E and M.F designed the study. S.R collected the data, performed the analysis and wrote the manuscript. M.H contributed to the interpretation of the results and work on the manuscript. F.M and P.P commented on the manuscript. All authors contributed to different versions of the manuscript and discussed the results and gave final approval for its publication.

## Data Availability

Data available from the Dryad Digital Repository: https://doi.org/10.5061/dryad.q4b18k3
